# Gut microbial alterations associated with the exacerbation of experimental autoimmune uveitis in PGRN-deficient mice

**DOI:** 10.3389/fimmu.2026.1641755

**Published:** 2026-01-23

**Authors:** Wenjun Zhou, Song Zhang, Chaokui Wang

**Affiliations:** 1 Department of Ophthalmology, The Affiliated Yongchuan Hospital of Chongqing Medical University, Chongqing, China; 2 State Key Laboratory of Pollution Control and Resource Reuse, School of the Environment, Nanjing University, Nanjing, China; 3 Ophthalmology Medical Center, The First Affiliated Hospital of Chongqing Medical University, Chongqing Key Laboratory for the Prevention and Treatment of Major Blinding Eye Diseases, Chongqing Branch (Municipality Division) of National Clinical Research Centre for Ocular Diseases, Chongqing, China

**Keywords:** 16S rRNA gene sequencing, autoimmune, gut microbiome, progranulin, uveitis

## Abstract

**Purpose:**

Progranulin (PGRN) has been shown to play a protective role in the development of a variety of immune-mediated diseases, and the gut microbiome has been implicated in the pathogenesis of autoimmune diseases. In this study, we investigate the changes in the gut microbiota and their association with the severity of experimental autoimmune uveitis (EAU) in PGRN-deficient mice.

**Methods:**

WT and PGRN-deficient C57BL/6 mice were used to induce EAU using interphotoreceptor-binding protein peptide. Gastrointestinal (GI) contents collected from both groups of induced EAU were subjected to 16S rRNA gene sequencing analysis.

**Results:**

PGRN-deficient mice developed exacerbated EAU compared to wild-type (WT) mice. The microbial richness of the GI contents in PGRN-deficient EAU mice was significantly lower than in WT mice. The PGRN-deficient EAU mice showed a significantly reduced microbial abundance in five phyla, namely, *Cyanobacteria*, *Epsilonbacteraeota*, *Firmicutes*, *Nitrospirae*, and *Patescibacteria*, and a significantly increased abundance in the other four phyla, namely, *Deferribacteres*, *Proteobacteria*, *Spirochaetes*, and *Tenericutes*. More importantly, a newly emerged phylum named *Chlamydiae* was detected in the gut microbial community of PGRN-deficient EAU mice. The histopathological scores were significantly negatively correlated with gut microbial abundance and significantly positively correlated with chlamydial abundance.

**Conclusion:**

Our results showed that PGRN plays a protective role in EAU, and the significant changes in the gut microbiome may be associated with the exacerbation of inflammation in the PGRN-deficient EAU mice.

## Introduction

PGRN binds competitively to tumor necrosis factor receptor (TNFR) and is therefore considered to be an endogenous antagonist of TNF-α, and it has been shown to play a protective role in a variety of autoimmune diseases, including inflammatory bowel disease (1), osteoarthritis (2), and rheumatoid arthritis (3). However, PGRN has also been shown to play a pathogenic role in some disease models, including diabetes mellitus and lupus nephritis (4, 5). In our study, we investigated the role of PGRN in experimental autoimmune uveitis (EAU), which shares clinical and immunopathological features with human uveitis. EAU is a classical animal model for the study of human uveitis and systemic autoimmune diseases. It can be induced in susceptible mouse strains (e.g., C57BL/6) by immunizing these mice with specific retinal antigens and adjuvants. Th1 and Th17 cells are thought to be effector immune cells that can induce or exacerbate inflammation, whereas Treg cells are thought to be a regulatory immune cell subset that can suppress it in this model (6).

Gut microbiota alterations critically regulate the immune system and influence disease severity in autoimmune models (7)—for example, patients with Behcet’s disease (BD) have a distinct gut microbiome signature compared to healthy controls, and the fecal transplantation into mice using feces from BD patients could exacerbate the disease activity and lead to an excessive production of pro-inflammatory cytokines (8). The EAU mice showed an altered microbiome compared to the non-immunized mice, and the clustering of gut microbial diversity was associated with clinical severity (9). Depletion or reduction of gut microbiota, particularly before disease induction, protects mice from severe EAU. This protection correlates with diminished retinal immune cell infiltration, reduced inflammatory T cell responses, and enhanced regulatory T cell populations in draining lymph nodes, indicating that microbiota modulates autoimmunity by shaping adaptive immunity during autoantigen recognition. Furthermore, specific probiotics can control inflammation in EAU (10, 11). Similar findings in experimental autoimmune encephalomyelitis (EAE) demonstrate that commensal microbiota are necessary for autoimmune demyelination (12), and multiple sclerosis patients have an altered intestinal microbiota compared to healthy controls (13). Collectively, these findings underscore the critical role of gut microbiota in autoimmune pathogenesis.

Preliminary work by our team has shown that PGRN could significantly reduce EAU severity and could inhibit IRBP161–180-specific Th1 and Th17 cell response and promote Treg cell expansion *in vivo* and *in vitro* (14). However, the exact mechanisms by which PGRN exert its protective role remains unclear. The gut microbiome has been considered to be implicated in the pathogenesis of autoimmune diseases. Therefore, we investigated whether there was a significant change in the gut microbiome in PGRN-deficient EAU mice and the association of the changes of gut microbiome with the severity of EAU.

## Methods

### EAU induction

PGRN-deficient mice of C57BL/6 background were purchased from Jackson Laboratory. All mice were maintained in a specific pathogen-free facility. Animal experimental protocols were approved and performed under the Animal Care and Use Committee of the Chongqing Medical University. EAU was induced as previously described. Briefly, WT and PGRN-deficient mice (*n* = 5) were immunized subcutaneously with 200 μL emulsion of 400 μg human IRBP651-670 (LAQGAYRTAVDLESLASQLT) in an equal volume of CFA containing 5 mg/ml *Mycobacterium tuberculosis* strain. Additionally, these mice also received a 1-μg intraperitoneal injection of *Bordetella pertussis* toxin (PTX, Sigma-Aldrich, St. Louis, MO, USA) (15). The experiments were conducted using 6–8-week-old female mice.

For clinical grading of EAU, the right eyes from both groups were examined by slit lamp microscopy on day 13 after immunization, and the clinical severity of inflammation was graded by two independent ophthalmologists in a masked fashion according to previously described criteria (16, 17). For histologic grading of EAU, the right eyes from both groups were enucleated, fixed, embedded in paraffin, sectioned, and stained with hematoxylin–eosin (H&E) on day 13 after immunization. Histological changes were graded by two independent observers in a masked fashion according to Caspi’s criteria (18).

### NovaSeq sequencing and bioinformatic analysis

16S rRNA gene sequencing analysis was conducted on fecal samples from four mice per group, which were randomly selected from the same cohort. The V3–V4 region of the 16S rRNA gene was amplified using PCR with universal primer sets 338F-806R. The PCR products were purified using the Axyprep DNA Gel extraction kit (Axygen, USA). Finally, Illumina NovaSeq high-throughput sequencing was performed. The raw pair-end sequences were spliced, and then the low-quality sequences with quality scores <20 and primers were removed using USEARCH (v11) (19). Operational taxonomic unit (OTU) representative sequences were generated using UNOISE3 with non-clustering denoise algorithm, and the OTU with a sequence number that represented less than 9 was removed (19). The OTU table was generated by the “otutab” command in USEARCH (v11). The taxonomic identities of representative OTU sequences were determined based on the SINTAX algorithm according to the Silva (v 138) database (20). The rooted phylogenetic trees were constructed by using the “phylogeny” command in QIIME2 (21). Finally, a total of 321 OTUs for prokaryotes was obtained. After being rarefied to the minimum sequence number in the samples, 49,995 sequences for bacteria were obtained in each sample. Microbial richness and Shannon index were estimated in R with the vegan package.

### Statistical analysis

All data were expressed as means ± SEM. Differences in the clinical and histological score were determined using Wilcoxon rank-sum test. Differences in microbial diversity between different groups were determined using Wilcoxon rank-sum test. The normality and homogeneity of variance of all the data were tested using Shapiro and Levene’s test, respectively. For the *β* taxonomic diversity, a Jaccard distance matrix at the OTU level among the samples was first created and analyzed by principal coordinates analysis (PCoA). The community composition dissimilarity matrix was calculated based on Bray–Curtis distance. Generalized linear model (GLM) was used to analyze the relationship between microbial richness and the severity of EAU. All of the statistical analyses were performed based on the ggpur, vegan, psych, reshape2, and packages in R.

## Results

### An exacerbation of EAU in PGRN-deficient mice

**Figure 1:**
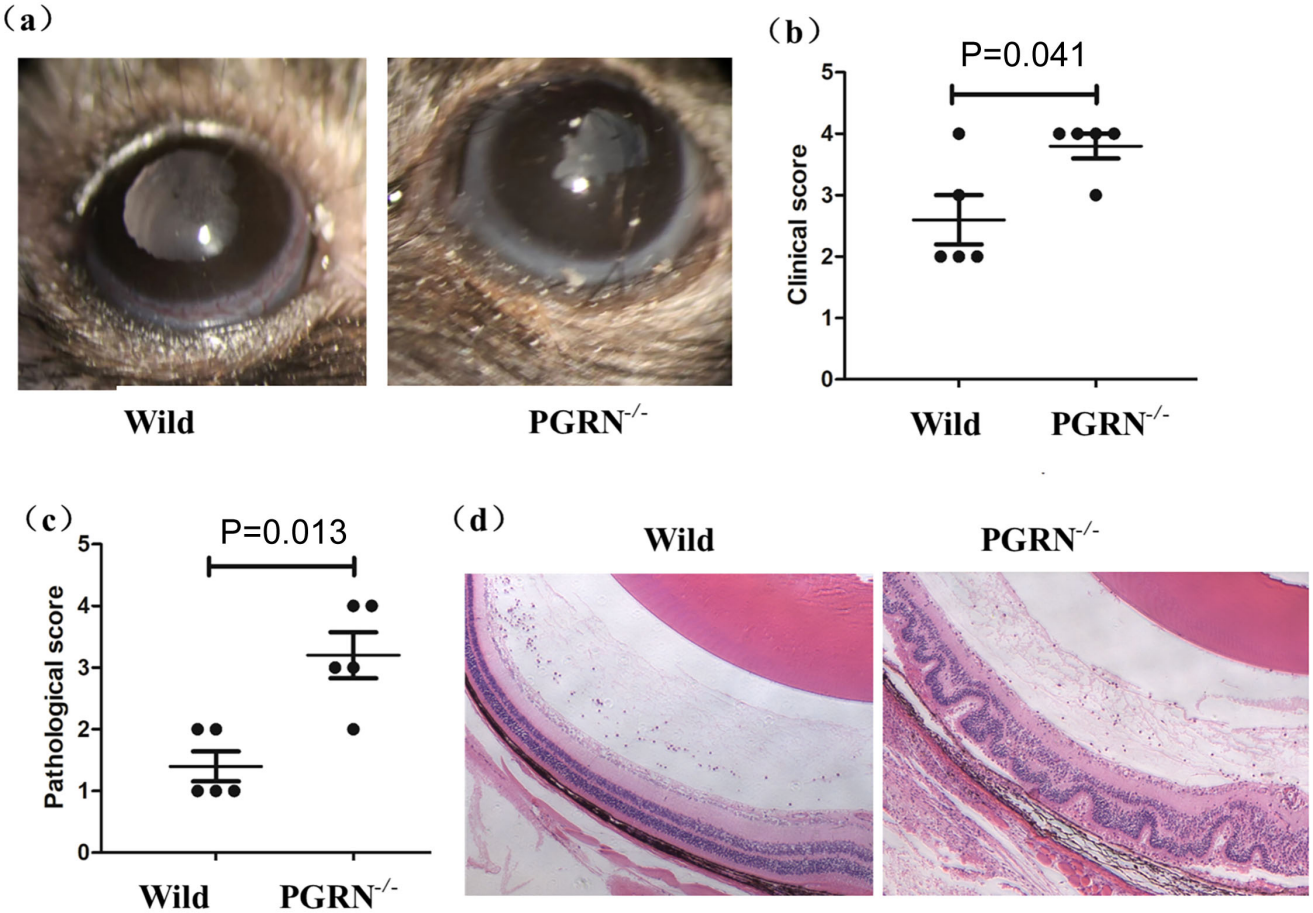
PGRN-deficient mice exhibit exacerbated EAU. WT and PGRN-deficient mice of C57BL/6 background (*n* = 5) were induced for EAU. The right eye of each mouse was used for analysis. **(a, b)** PGRN-deficient mice had significantly higher clinical scores than WT controls. **(a)** Representative slit-lamp section images of the two groups on day 13 after immunization. **(b)** The clinical scores of the two groups were measured on day 13. Wilcoxon rank-sum test was used for the statistical analysis (*P* < 0.05, error bars, SD). **(c, d)** The pathologic scores were significantly higher in the PGRN-deficient mice than those in WT controls. **(c)** The pathologic scores of the two EAU groups were measured on day 13. Wilcoxon rank-sum test was used for the statistical analysis (*P* < 0.05, error bars, SD). **(d)** Representative H&E-stained sections of EAU eyes on day 13 after immunization. Scale bar, 30 µm. EAU, experimental autoimmune uveitis; PGRN-/-, PGRN-deficient; wild, wild type.

To investigate the role of PGRN in EAU, we induced uveitis in WT and PGRN-deficient mice on a C57BL/6 background. Clinical observations revealed that PGRN-deficient mice exhibited significantly higher scores compared to WT controls (**Figures 1a, b**). Histologically, WT mice showed moderate chorioretinal lesions and cellular infiltration within the eyes. However, PGRN-deficient mice displayed more severe chorioretinal lesions, pronounced inflammatory cellular infiltration, and extensive retinal folding. The pathologic scores were significantly higher in the PGRN-deficient mice than those in WT controls (**Figures 1c, d**).

### Changes in microbial diversity and community composition in PGRN-deficient EAU mice

**Figure 2:**
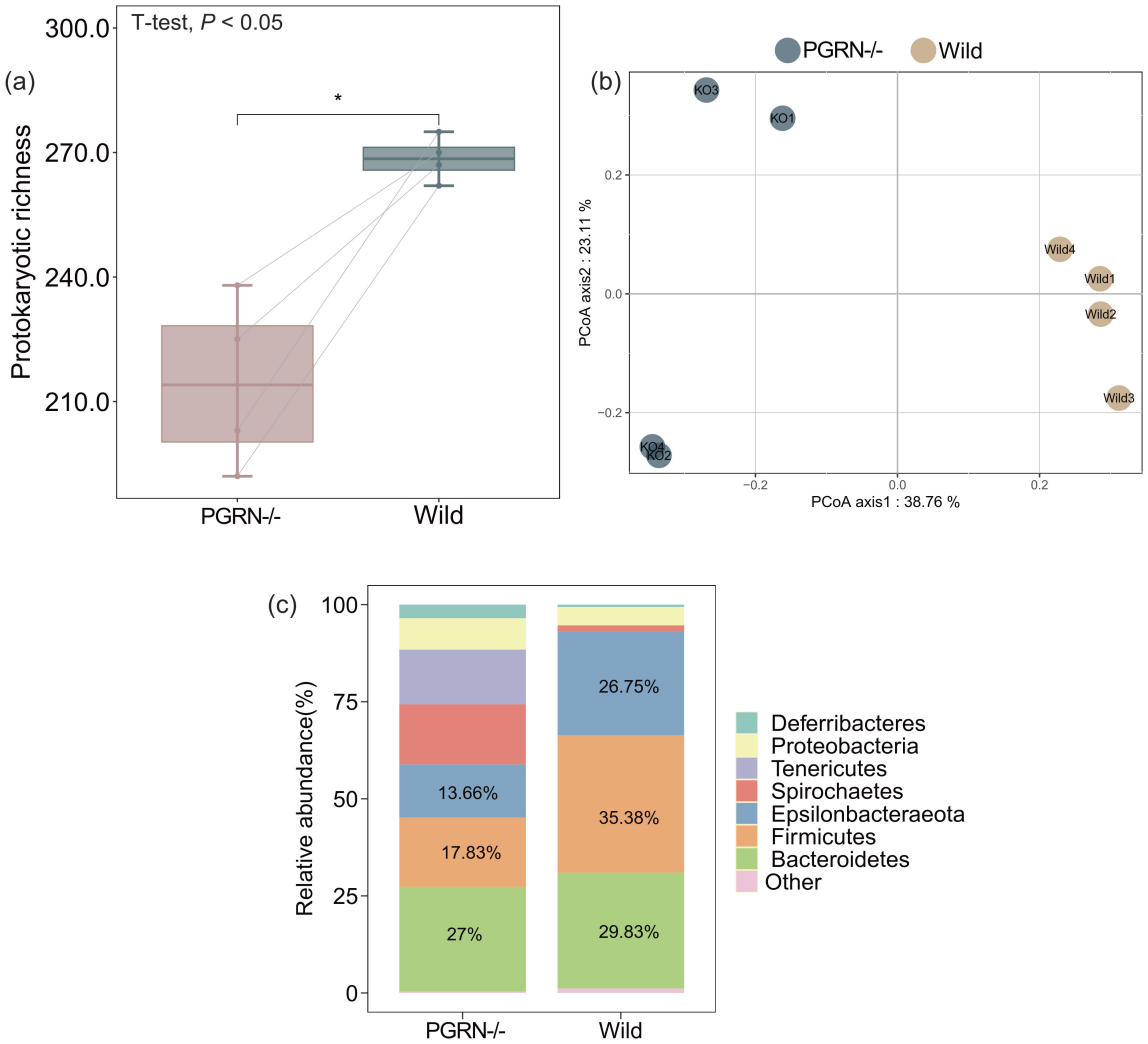
Significant differences in the microbial diversity and community composition between WT and PGRN-deficient EAU mice. **(a)** The microbial diversity, as indicated by alpha diversity measured with the Shannon index, was significantly reduced in PGRN-deficient EAU mice compared to WT controls. Wilcoxon rank-sum test was used for the statistical analysis (*P* < 0.05). **(b)** PCoA ordinations of prokaryotic community profiles revealed a significant difference between the two groups (*P* = 0.001). **(c)** The overall community composition of prokaryotes differed between WT and PGRN-deficient EAU mice. The percentage of the three dominant phyla, including Firmicutes, Bacteroidetes, and Epsilonbacteraeota, was reduced in PGRN-deficient mice. EAU, experimental autoimmune uveitis; PGRN-/-, PGRN-deficient; wild, wild type.

To explore the gut microbiota changes associated with the exacerbation of EAU in PGRN-deficient mice, we employed 16S rRNA gene sequencing analysis. Our results indicated a significant reduction in intestinal microbial diversity in PGRN-deficient EAU mice compared to their WT counterparts (Wilcoxon rank-sum test, *P* < 0.05; **Figure 2a**). Furthermore, the PCoA diagram revealed a pronounced difference in microbial community composition between WT and PGRN-deficient EAU mice (PERMANOVA, *P* = 0.001; **Figure 2b**). At the phylum level, the intestinal microbial community of WT EAU mice was predominantly composed of three phyla—*Firmicutes*, *Bacteroidetes*, and *Epsilonbacteraeota*—which collectively accounted for 91.96% of the community (**Figure 2c**). In contrast, in PGRN-deficient EAU mice, the combined percentage of *Bacteroidetes*, *Firmicutes*, and *Epsilonbacteraeota* dropped to 58.49%, with *Spirochaetes* and *Tenericutes* emerging as the next most prevalent phyla in the gut microbial community (**Figure 2c**).

### Changes in microbial phylum level in PGRN-deficient EAU mice

**Figure 3:**
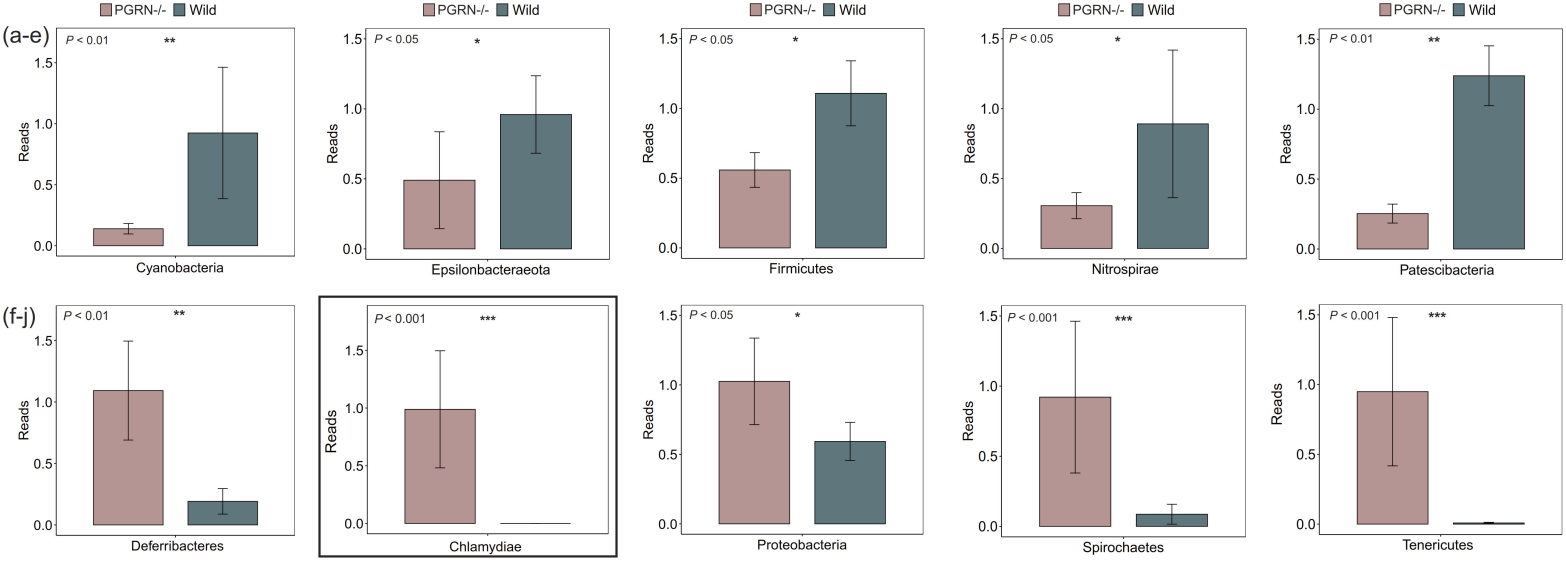
Microbial phylum-level differences between PGRN-deficient EAU and WT EAU mice. A total of 10 phyla (five upregulated and five downregulated) were identified as significantly different between PGRN-deficient EAU and WT EAU mice. **(a–e)** The abundance of Cyanobacteria, Epsilonbacteraeota, Firmicutes, Nitrospirae, and Patescibacteria was decreased in PGRN-deficient EAU mice. **(f–j)** The abundance of Deferribacteres, Proteobacteria, Spirochaetes, and Tenericutes was significantly increased. Additionally, the Chlamydiae phylum was newly detected in the intestinal microbial community of PGRN-deficient EAU mice. Wilcoxon rank-sum test was used for the statistical analysis. EAU, experimental autoimmune uveitis; PGRN-/-, PGRN-deficient; WT, wild type. *, P<0.05; **, P<0.01; ***, P<0.001.

We analyzed the differences in microbial phylum levels between PGRN-deficient EAU and WT EAU mice using Wilcoxon rank-sum test. Our analysis identified a total of 10 significantly different phyla, with five upregulated and five downregulated, in PGRN-deficient EAU mice compared to WT EAU mice (**Figure 3**). Specifically, the abundance of *Cyanobacteria*, *Epsilonbacteraeota*, *Firmicutes*, *Nitrospirae*, and *Patescibacteria* was significantly lower in PGRN-deficient EAU mice (**Figures 3a–e**, *P* < 0.05). Conversely, the abundance of *Deferribacteres*, *Proteobacteria*, *Spirochaetes*, and *Tenericutes* was significantly higher in PGRN-deficient EAU mice. Interestingly, a newly detected phylum, identified as *Chlamydiae*, was present in the intestinal microbial community of PGRN-deficient EAU (which comprised approximately 1% of the total microbiome), but it was absent in WT EAU mice (**Figures 3f–j**, P < 0.05).

### Association of microbial richness and *Chlamydiae* abundance with EAU severity

**Figure 4:**
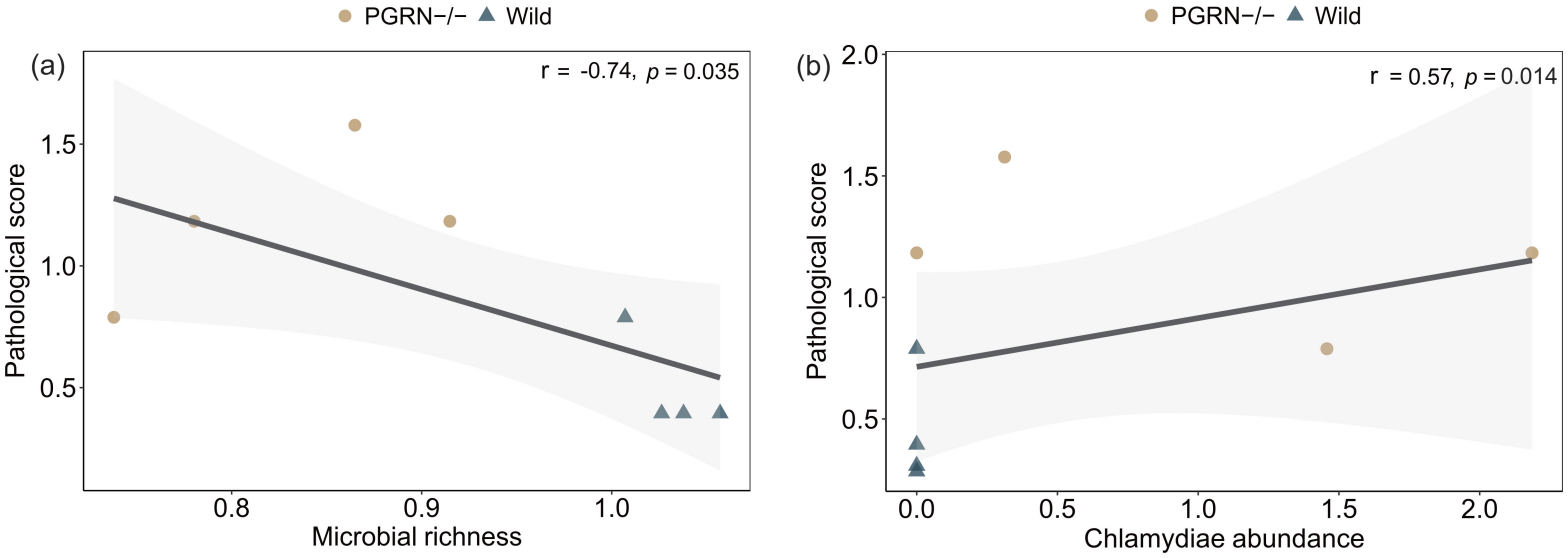
Association of microbial changes with the severity of EAU. **(a)** Negative association between the microbial richness and the histopathological score of EAU (*r* = -0.74, *P* = 0.035). **(b)** Positive association between the Chlamydiae abundance and EAU histopathological score (*r* = 0.57, *P* = 0.014). Each point (including circles and triangles) represents an individual mouse. Generative language model (GLM) was used for the statistical analysis.

We examined the correlation between changes in microbial richness and *Chlamydiae* abundance with the histopathological score using GLM analysis. Our analysis revealed a significant negative correlation between gut microbial richness and the histopathological score (**Figure 4a**; *r* = -0.74, *P* < 0.05). Additionally, we identified a significant positive correlation between the abundance of the newly detected *Chlamydiae* phylum in PGRN-deficient EAU mice and the pathological score (**Figure 4b**; *r* = 0.57, *P* < 0.05).

## Discussion

In the present study, we evaluated the impact of PGRN deficiency on experimental autoimmune uveitis (EAU) and investigated the gut microbiota changes in the induced uveitis in PGRN-deficient mice. Our data showed that PGRN deficiency could lead to an exacerbated EAU and distinct alterations to the gut microbiota. These changes to the gut microbiota may be associated with the exacerbation of inflammation in PGRN-deficient mice.

In our study, we found that PGRN deficiency could exacerbate the severity of EAU by comparing the severity of inflammation in WT and PGRN-deficient EAU mice. Previous studies have shown that it plays a protective role in some autoimmune mouse models, such as collagen-induced arthritis, colitis, and osteoarthritis (3, 22, 23). However, PGRN mediates pro-inflammatory immune responses in systemic lupus erythematosus and in some inflammatory diseases, such as the pulmonary immunopathology during influenza virus infection and the lethal *Candida albicans* (24–26). Collectively, these results suggest that PGRN exerts different roles in different immune or inflammatory conditions and that it exerts an important protective benefit in EAU.

In this study, we found that PGRN-deficient EAU mice exhibited significantly reduced gut microbial species richness and an altered community composition compared to wild-type (WT) EAU controls. In contrast, Gu et al. reported that PD-L1 deficiency increased microbial richness and modulated key bacterial families (*Bacteroidaceae*, *Lachnospiraceae*, and *Ruminococcaceae*) (27). These results suggest that PGRN may play a role in maintaining microbiota diversity, and that the decrease in microbial diversity observed in PGRN-deficient EAU mice may be associated with exacerbated uveitis. Our findings are in line with previous reports showing a decreased microbial diversity in the rheumatoid arthritis patients compared to healthy controls and the decreased species richness was associated with the rheumatoid factor levels and disease duration (28, 29). However, our previous reports did not find a statistically significant difference in the α- diversity or β-diversity of species abundance between the uveitis patients with Vogt-Koyanagi-Harada (VKH) disease and normal controls (30). This difference may be due to the significant individual differences or to the small number of individuals in each category. In summary, these findings indicate an association between microbial diversity and the severity of autoimmune disease, studies are needed to establish the causality in further. We next analyzed the specific phylum-level alterations in the microbial composition involved in the exacerbation of EAU in PGRN-deficient mice. At the phylum level, the PGRN-deficient EAU mice showed a significant decrease in the proportion of *Cyanobacteria*, *Epsilonbacteraeota*, *Firmicutes*, *Nitrospirae*, and *Patescibacteria*. Our results are consistent with previous reports showing that *Firmicutes*, *Nitrospirae*, and *Cyanobacteria* were negatively associated with disease development. *Firmicutes* and *Cyanobacteria* are known to be the predominant bacteria in normal human gut (31). The instability or decrease in the number of *Firmicutes* and *Cyanobacteria* is referred to as “microbial dysbiosis” (32). Patients with rheumatoid arthritis had a reduced proportion of the *Firmicutes* phylum compared to normal controls (29). Patients with systemic lupus erythematosus had reduced levels of the *Nitrospirae* phylum (33). Regarding cyanobacteria, Zhu et al. reported that cyanobacterial abundance was significantly lower in patients with allergic rhinitis compared to the controls (34). These results suggest that *Firmicutes*, *Nitrospirae*, and *Cyanobacteria* are responsible for maintaining the micro-ecological balance, and the decrease in these microbial compositions was associated with the exacerbation of EAU. However, increased levels of *Epsilonbacteraeota* and *Patescibacteria* were observed in patients with osteoarthritis and ulcerative colitis, respectively (35, 36). These discrepancies suggest that each disease has a unique microbiome composition.

In our study, we observed that PGRN-deficient EAU mice exhibited a significantly higher proportion of *Deferribacteres*, *Proteobacteria*, *Spirochaetes*, and *Tenericutes*. Notably, our findings relating to *Proteobacteria* are consistent with those of a previous study on ulcerative colitis, which showed the increased relative abundance of *Proteobacteria* at the phylum level in patients with active ulcerative colitis compared to healthy controls. Therefore, these two studies therefore suggest that *Proteobacteria*, which are normally minor phylum, increase in prevalence in these diseases (36). Besides these increased microbial compositions, we identified a new *Chlamydiae* phylum in PGRN-deficient EAU mice and found that *Chlamydiae* abundance was positively associated with EAU severity. *Chlamydiae* are Gram-negative bacteria with pathogenic potential for a wide variety of diseases. *Chlamydia trachomatis*, which represents the main human *Chlamydia* pathogenic species, is responsible for ocular infection (37). It has been reported that three types of antibodies to *Chlamydiae* are significantly more prevalent in patients with ocular inflammation than in healthy controls (38). Our sequencing data reflects gastrointestinal colonization. We therefore hypothesize that PGRN deficiency increases susceptibility to intestinal chlamydial colonization, which could indirectly exacerbate autoimmunity through molecular mimicry or generalized immune activation. These findings demonstrate an association between chlamydial abundance and EAU severity in PGRN-deficient mice. However, further studies are needed to clarify the causal relationship.

Our study is limited by the evaluation of only one time point and the small sample size, which make it challenging to draw definitive conclusions. However, we believe that our findings contribute to the growing body of literature on the gut microbiome and its potential role in autoimmune diseases. Further studies are needed to explore the relationship between PGRN, gut microbiota, and EAU.

In conclusion, our results demonstrate that PGRN exerts a protective role in EAU, and there was a significant alteration in the gut microbiota which may be associated with the severity of inflammation in PGRN-deficient EAU mice.

## Data Availability

The raw data supporting the conclusions of this article will be made available by the authors, without undue reservation.
